# International variance in the treatment of developmental dysplasia of the hip

**DOI:** 10.1007/s11832-014-0622-z

**Published:** 2014-10-29

**Authors:** I. H. Feeley, C. J. Green, F. E. Rowan, D. P. Moore

**Affiliations:** Our Lady’s Children’s Hospital, Crumlin, Dublin 12, Ireland

**Keywords:** Developmental dysplasia of the hip, Paediatrics, Treatment, Consensus

## Abstract

**Introduction:**

Developmental dysplasia of the hip (DDH) is the most common congenital musculoskeletal abnormality. Recourse to definitive surgical treatment is not typically taken until over the age of 18–24 months. International consensus regarding age at surgery, degree of dysplasia requiring surgery and type of osteotomy is not available in the literature.

**Study aims:**

To determine variation in timing and type of osteotomy for persistent DDH across the world.

**Methodology:**

Senior authors of recent publications pertaining to hip dysplasia were sampled. Participants’ practice relating to age and radiological indications for surgery were determined.

**Results:**

Thirty-two surgeons responded from five different geographical regions. No inter-regional consensus was established regarding investigations to determine the need for osteotomy, preferred osteotomy type or ideal age at which to perform an osteotomy.

**Conclusion:**

International agreement regarding the surgical management of DDH does not exist. This common congenital condition warrants development of a treatment algorithm.

## Introduction

Developmental dysplasia of the hip (DDH) is a common musculoskeletal condition, referring to a spectrum of abnormalities of the hip joint, encompassing a range of abnormal morphologies from frank dislocation to hip dysplasia [1]. The natural history of the condition is variable: children with initially abnormal examination or radiological findings can resolve spontaneously, without the need for surgery [2]. The estimated incidence of the condition varies widely (1.5–20 per 1,000 births) [3, 4], reflecting the spectrum of abnormalities under the heading of DDH. Consensus is also lacking in the screening regimes of neonates, contributing to the wide variance in incidence [5].

Treatment is initially conservative, with the use of braces, harnesses or spica casts [6, 7]. Persistent dysplasia of the acetabulum may require a pelvic osteotomy [8]. The ideal age range for this intervention is not established in the literature [9], although leaving intervention beyond the age of 8 years has been established as non-advantageous [8]. The literature equally does not offer straightforward evidence concerning the osteotomy type to be applied, with publications advocating the merits of various eponymous procedures [10–12].

No international guidelines or algorithm exist for surgical or non-surgical approaches to the management of DDH [13]. Our study aims to establish whether a consensus exists among paediatric orthopaedic surgeons regarding the timing and type of operative treatment of residual DDH.

## Methods

A search of the pertinent literature was carried out on the PubMed search engine. This search yielded 92 senior authors who were invited to partake in the study. Five geographical regions were determined: Europe, North America, Asia, Australasia and rest of the world (RoW). Participants were sampled by electronic questionnaire using the survey tool SurveyMonkey (http://www.surveymonkey.com). Questions were asked in the context of a stable reduced hip with residual dysplasia in a child aged 18–36 months. The survey focused on four aspects of care: investigations prior to osteotomy, factors affecting decision for osteotomy, preferred osteotomy type and post-operative immobilisation. The scenario was designed to serve as a template by which respondents could express their preferences for these four aspects of care. Descriptive statistics were applied.

Approval from the institutional review board was not required for this study.

## Results

There were 32 respondents from the five regions: North America (*n* = 7; five institutions), Europe (*n* = 14; 13 institutions), China and Japan (*n* = 3; three institutions), Australia and New Zealand (*n* = 5; four institutions) and RoW (*n* = 3; three institutions). Of the respondents, 81 % were fellowship trained in paediatric orthopaedics. The results are shown in tabular form in Table 1.

**Table 1:** Survey questions and results

Question	Options								
1: I use the following investigations in determining the need for osteotomy	X-ray	CT	MRI	Arthrogram					
Results (%)	94	13	9	38					
2: I use the following measurements in determining the need for osteotomy	CHDD	AI	CEA	Sourcil angle	Morphology	Other			
Results (%)	19	81	34	19	47	16			
3: I make my decision for osteotomy at age	18 months	2 years	2.5 years	3 years	3.5 years	4 years	4.5 years	5 years or over	Do not consider age
Results (%)	23	7	13	17	13	10	7	10	6
4: I would not defer an osteotomy which had persistent dysplasia beyond the age of	Never beyond 2 years	2.5 years	3 years	3.5 years	4 years	4.5 years	5 years or more	Do not consider age	
Results (%)	16	0	9	0	19	9	44	3	
5: I am more inclined to perform an osteotomy if there is	Family history	Hip unstable at diagnosis	Late presentation	Open reduction required	Bilateral disease	Other			
Results (%)	12	19	19	22	6	3			
6: My osteotomy of choice is	Based on the X-ray	Based on MRI	Based on arthrogram	A Salter osteotomy regardless	A Pemberton osteotomy regardless	A Dega osteotomy regardless	Other		
Results (%)	34	3	28	19	9	6	0		
7: Post-osteotomy, I preferentially apply a	Spica cast	Bachelor cast	Abduction orthosis	Thomas splint	Nothing				
Results (%)	88	0	6	0	6				

The first question concerned the imaging modalities typically used to determine the need for pelvic osteotomy. Pelvic radiographs are used by 94 % of respondents. Less than half (38 %) use hip arthrography. Few surgeons use advanced imaging modalities—either computed tomography (CT) (13 %) or magnetic resonance imaging (MRI) (9 %). North American and RoW respondents use only a plain pelvic radiograph, with none of these respondents using hip arthrography or three-dimensional (3D) imaging modalities.

In Australasia, all respondents use plain pelvic radiographs and 66 % augment this with arthrography. Of the European respondents, 8 % do not use plain film radiographs, 62 % use arthrogram and a low number use CT and MRI (23 and 15 %, respectively). CT and MRI are also used by 33 % of Asian respondents, 67 % of whom use both plain film and arthrogram. The authors’ preference is to use X-ray, CT and arthrogram to determine the need for an osteotomy.

Question two examined the radiological indices used by surgeons when interpreting hip imaging. These were (frequency): centre head distance discrepancy (CHDD) (19 %), acetabular index (AI) (81 %), centre edge angle (CEA) (34 %), sourcil angle (19 %) or morphology appearance (47 %). There is a broad spread across all regions for the radiological indices used (Table 2). The authors’ preferred indices are CHDD, AI and CEA.

**Table 2:** Radiological indices by region (percentage)

Region	North America	Australia and New Zealand	Asia	Europe	RoW
CHDD	0	20	0	31	33
AI	75	80	100	85	67
CEA	0	80	33	31	33
Sourcil angle	25	20	0	23	0
Morphology	63	40	33	46	33

The third question concerned the age at which decision for osteotomy was taken. Options were from 18 months to 5 years or over. Overall, 6 % do not use age as a criterion for performing an osteotomy. Twenty-three percent of respondents operate at 18 months. Beyond 18 months, 7 % intervene at 2 years, 13 % at 2.5 years, 17 % at 3 years, 13 % at 3.5 years, 10 % at 4 years and 7 % at 4.5 years. The remaining 10 % stated that they would perform an osteotomy aged 5 years or older. Of North American surgeons, 87 % answered one of the options between 3 years and 4.5 years; 80 % of Australasians intervened between 18 months or 2 years. All RoW respondents answered that they perform an osteotomy by the age of 3 years. European and Asian respondents were evenly distributed over the age ranges given, and 8 % of European surgeons did not use age as a criterion to perform osteotomy. The authors’ preferred age to make a decision on osteotomy is 2.5 years.

Question four asked what is the upper age limit for deferring an osteotomy in a child with residual dysplasia. Overall, 44 % are willing to defer a child to the age of 5 years or beyond, while 16 % stated that they do not delay an osteotomy beyond the age of 2 years. All North American respondents state that they are willing to defer an osteotomy until at least the age of 4 years. Of the European surgeons, 23 % do not defer beyond 2 years and 61 % would wait until 4.5 years or over. Of the Australasian surgeons, 40 % use 3 years as their cut-off, 40 % choose 4 years and 20 % choose 5 years or over. For the Asian and RoW responders, 33 % said never beyond the age of 2 years, 67 % of RoW would defer surgery to 4 years and 67 % of Asian surgeons choose 5 years or over. The author would be unwilling to defer surgery beyond 3 years.

**Fig. 1:**
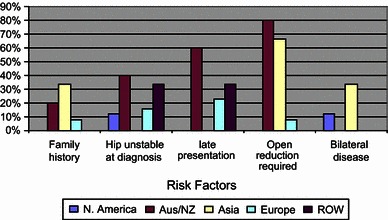
Question five by region

Question five examined the impact of patient factors in the decision for performing an osteotomy. The options and responses were: family history of DDH (21 %), late presentation of over 4 months (32 %), bilateral DDH (32 %), unstable hips at diagnosis (37 %) and the need for open reduction (11 %). Some 59 % of respondents stated that their decision for surgery would take account of at least one of these factors. Overall, 54 % of Europeans use at least one of these as a contributing factor for a decision to perform an osteotomy, 33 % of the ROW cohort, 100 % of Asian and Australasian surgeons and 50 % of North American responders. Figure 1 shows the response to question five by region. The author is more inclined to perform an osteotomy if the presentation is late.

The type of osteotomy preferred by surgeons is the focus of question six. The type of osteotomy is determined by plain pelvic radiograph by 34 % of respondents and by arthrography by 28 % of respondents. Overall, 34 % state that morphology has no impact on the choice of osteotomy performed. Just one responder stated that they use MRI to determine osteotomy choice. Of the Europeans, 31 % based osteotomy on pelvic radiographs and 46 % on arthrogram. Twenty-three percent of European respondents perform a Salter osteotomy regardless of acetabular morphology or imaging. In North America, 63 % base their decision on pelvic radiographs, while 37 % perform a Pemberton or Dega osteotomy regardless of acetabular morphology or imaging. The majority of Australasian respondents (60 %) use arthrography to decide on the osteotomy type, while 40 % choose either a Salter or a Pemberton osteotomy. In Asia, 33 % use pelvic radiographs, 33 % use MRI and 33 % perform a Salter osteotomy regardless of acetabular morphology or imaging. RoW respondents were also split: 66 % perform an eponymous osteotomy regardless of acetabular morphology or imaging and 33 % use pelvic radiographs. The osteotomy of choice for the author is based on the arthrogram.

Question seven asked about post-operative immobilisation. Spica cast immobilisation is the most commonly used, by 88 % of respondents. Alternative choices from respondents were the use of a hip abduction brace and no immobilisation of patients (6 % of respondents for each). Every respondent from outside of Europe used a spica cast, while within Europe, 15 % use an abduction brace and 15 % use no post-operative immobilisation. The author uses a spica cast post-operatively.

The institutions represented in the survey are shown in Table 3.

**Table 3:** Institutions represented in survey

Europe	University Hospital, Düsseldorf South Infirmary Victoria University Hospital, Cork Temple Street University Hospital, Dublin Our Lady’s Children’s Hospital, Dublin Inselspital, Bern Bristol Royal Hospital for Children East Kent Hospitals University NHS Foundation Trust Queen Alexandra Hospital, Portsmouth Great North Children’s Hospital, Newcastle Robert Jones and Agnes Hunt (RJAH) Orthopaedic Hospital, Oswestry Great Ormond Street Hospital, London
North America	Texas Scottish Rite Hospital for Children, Dallas The Hospital for Sick Children, Toronto Arnold Palmer Hospital for Children, Orlando Shriners Hospitals for Children, Montreal University of Iowa Hospitals and Clinics, Iowa City Riley Children’s Hospital, Indianapolis
Australasia	The Children’s Hospital at Westmead, Sydney Royal Children’s Hospital, Melbourne Royal Hobart Hospital, Tasmania Starship Children’s Hospital, Auckland
Asia	Shengjing Hospital of China Medical University, Shenyang, China Children’s Hospital of Fudan University, Shanghai, China Kyushu University Hospital, Japan
Rest of World	CURE Ethiopia Children’s Hospital, Ethiopia Ankara Numune Training and Research Hospital, Ankara, Turkey Metin Sabanci Baltalimani Education and Training Hospital, Istanbul, Turkey

## Discussion

The treatment of DDH is not governed by any international guidelines or algorithm. The variations in factors influencing treatment reflect the uncertainty surrounding the definition [3], best screening methods [4] and epidemiology [14] of the condition. Our study aimed to take a snapshot of current surgical treatment practices from orthopaedic colleagues around the world for the treatment of a dysplastic acetabulum with a stable reduced femoral head. The results demonstrate marked variation. This variation is evident between regions for some aspects of investigation prior to deciding osteotomy, but, otherwise, intra-regional differences are shown to exist.

To the authors’ knowledge, no previous studies have attempted to establish regional variations. Interestingly, the North American and RoW cohort utilise only pelvic radiographs to determine the need for osteotomy, whilst the arthrogram is popular in the other three regions. Few use the advanced imaging modalities of CT and MRI, the advantages to which are not fully established [15–17].

**Fig. 2:**
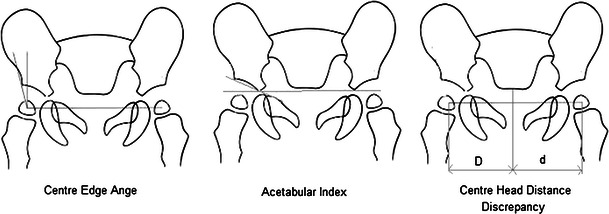
Indices used to assess developmental dysplasia of the hip (DDH) on plain radiographs. The centre edge angle (CEA) is the angle from the lateral wall of the acetabulum to the centre of the femoral head relative to the vertical. The acetabular index (AI) is the angle between Hilgenreiner’s line and a line drawn from the triradiate cartilage to the lateral edge of the acetabulum. The centre head distance discrepancy (CHDD) is the percentage difference between *D* and *d*

The radiological measurements that are available as options are each valid tools in the assessment of pelvic radiographs [18–20]. The reproducibility and reliability of these indices is not absolute [21] and, so, it is interesting, but not surprising, that 45 % of respondents still use morphology as an indication for surgery. The progression, or lack of progression, was a frequent comment in the other box. However, for this question, we were hoping to examine index use, or lack thereof, in deciding upon surgery. The indices are shown in Fig. 2. The sourcil angle is taken to be the angle from the lateral to medial aspect of the sourcil with respect to the horizontal.

The remodelling potential of the acetabulum has been shown to diminish around the age of 5 years, with Brougham et al. [22] finding a range of 17 months to 8 years for the cessation of development. The evidence in the literature is not conclusive regarding the optimum age for surgery for the correction of residual dysplasia [9, 23, 24], and no published consensus exists. This uncertainty as to best practice is borne out in our results, with no intra- or inter-regional pattern evident for preferred age for osteotomy.

There is no evidence in the literature for patient factors contributing in the decision to perform an osteotomy. This study shows that these patient factors are included in the decision-making matrix of 59 % of surgeons who responded. The eponymous osteotomies described in the literature used in this age group are the Dega osteotomy [10] and the Pemberton osteotomy [25], both of which hinge at the triradiate cartilage, and the Salter osteotomy [11, 24], which hinges at the pubic symphysis. Our survey has shown that 35 % of surgeons choose their osteotomy regardless of acetabular morphology or imaging. Regional variations are evident. The Australasian cohort did not use any imaging modality bar arthrography to decide osteotomy type. Europe was the only other region to use arthrogram to determine osteotomy type. Asian respondents were the only surgeons from any region that use an MRI to decide osteotomy type.

Post-operative immobilisation has traditionally been achieved with a spica cast [26]. This is borne out by our study, with 88 % of surgeons using this mode of immobilisation. Europe was the only region diverging from spica use. Fifteen percent of respondents from the region opt for hip abduction braces post-operatively, and a further 15 % do not use any post-operative immobilisation.

The limitations of this study are to be noted. Surveys are prone to responder bias. The sample was limited to the orthopaedic surgeons with whom we could establish contact, and although good worldwide variation was achieved, the overall numbers were not particularly high. Despite this, we expect that individual surgical philosophy often follows local procedure and, therefore, singular responses do indeed reflect institutional practice. Our response rate was 35 %. The level of detail that was asked is low; in this survey, it was felt that, in order to generate a response from time-poor individuals, succinct questions were required.

## Conclusion

The treatment of developmental dysplasia of the hip (DDH) is not subject to any international guidelines or consensus. This results in varied practice, both inter-regionally and intra-regionally. The topic is one which could be subjected to an international working group for a large-scale study of different techniques, allowing for the formation of an algorithm for the operative intervention of this common disorder.
